# Characterization of the complete mitochondrial genomes of two Critically Endangered wedgefishes: *Rhynchobatus djiddensis* and *Rhynchobatus australiae*

**DOI:** 10.1080/23802359.2023.2167479

**Published:** 2023-03-07

**Authors:** M. J. Groeneveld, J. D. Klein, R. H. Bennett, A. E. Bester-van der Merwe

**Affiliations:** a Department of Genetics, Molecular Breeding and Biodiversity Group, Stellenbosch University, Stellenbosch, South Africa; b Wildlife Conservation Society, Western Indian Ocean Shark and Ray Conservation Program, Bronx, NY, USA

**Keywords:** Ion Torrent, Rhinopristiformes, mitogenomes, phylogenetic analysis, rhino rays

## Abstract

We present the complete mitochondrial genomes of the Critically Endangered whitespotted wedgefish, *Rhynchobatus djiddensis* (Forsskål, 1775), and bottlenose wedgefish, *Rhynchobatus australiae* (Whitley, 1939), with the *R. djiddensis* mitogenome documented for the first time. The genomes for *R. djiddensis* and *R. australiae* are 16,799 and 16,805 bp in length, respectively. Both comprise 13 protein-coding regions, 22 tRNA genes, two rRNA genes, and a non-coding control region. All protein-coding regions consistently start with the ATG start codon; however, the alternative start codon GTG is observed at the start of the *COX1* gene. *NADH2*, *COX2*, and *NADH4* have incomplete stop codons: T or TA, and *tRNA^Leu^* and *tRNA^Ser^*, have atypical codons: UAA, UGA, GCU, and UAG. The phylogenetic analysis places *R. djiddensis* and *R. australiae* within the *Rhynchobatus* genus, separate from other families in the order Rhinopristiformes. We also highlight the most variable gene regions to expedite future primer design, of which *NADH2* was the most variable (4.5%) when taking gene length into account. These molecular resources could promote the taxonomic resolution of the whitespotted wedgefish species complex and aid in the genetic characterization of populations of these and related species.

## Introduction

Rhino rays (wedgefishes and giant guitarfishes) from the subclass Elasmobranchii are the most imperiled marine taxa in the world, with all but one of the 16 described species classified as Critically Endangered according to the International Union for Conservation of Nature (IUCN) Red List of Threatened Species, and all are listed in Appendix II of the Convention on International Trade in Endangered Species (CITES) (Choo et al. 2021; IUCN 2022). This is due to a combination of their K-selected life history traits, presence in shallow waters which intersect with coastal fisheries, and overexploitation through targeted and incidental catch, driven by the need for animal protein and the trade in their high-value fins (Kyne et al. 2020). Furthermore, significant taxonomic uncertainty is associated with the *Rhynchobatus* genus; thus, individuals recorded in e.g. fisheries landings are often synonymized as a single species complex referred to as the whitespotted wedgefish complex. Molecular taxonomic studies of rhino rays are mostly limited in scope to single genetic markers (Aschliman et al. 2012) which are not always accessible for the relevant species and do not enable fine-scale population genetic analyses. The lack of clear evolutionary significant units and molecular resources has compromised species-specific fishery and demographic data and further impedes assessments of conservation status, enforcement of laws and management of these highly threatened species (Henderson et al. 2016; Kyne et al. 2020). In particular, the whitespotted wedgefish, *Rhynchobatus djiddensis* (Forsskål, 1775), and the bottlenose wedgefish, *Rhynchobatus australiae* (Whitley, 1939), found across the Southwest Indian Ocean region have an extremely high risk of extinction and lack of baseline information (White et al. 2014; Kyne et al. 2020; Daly et al. 2021).

As such, the aim was to assemble and annotate the complete mitogenomes of *R. djiddensis* and *R. australiae* from high-throughput sequencing data and to infer the phylogenetic placement of these two species within the order Rhinopristiformes. We also highlight the most variable gene regions to aid with primer design and the amplification of alternative mitochondrial markers. Future studies can utilize the genomic resources developed here to refine species identification and genetic characterization in these and related species.

## Materials and methods

### Samples, DNA extraction, and high-throughput sequencing

**Figure 1.**
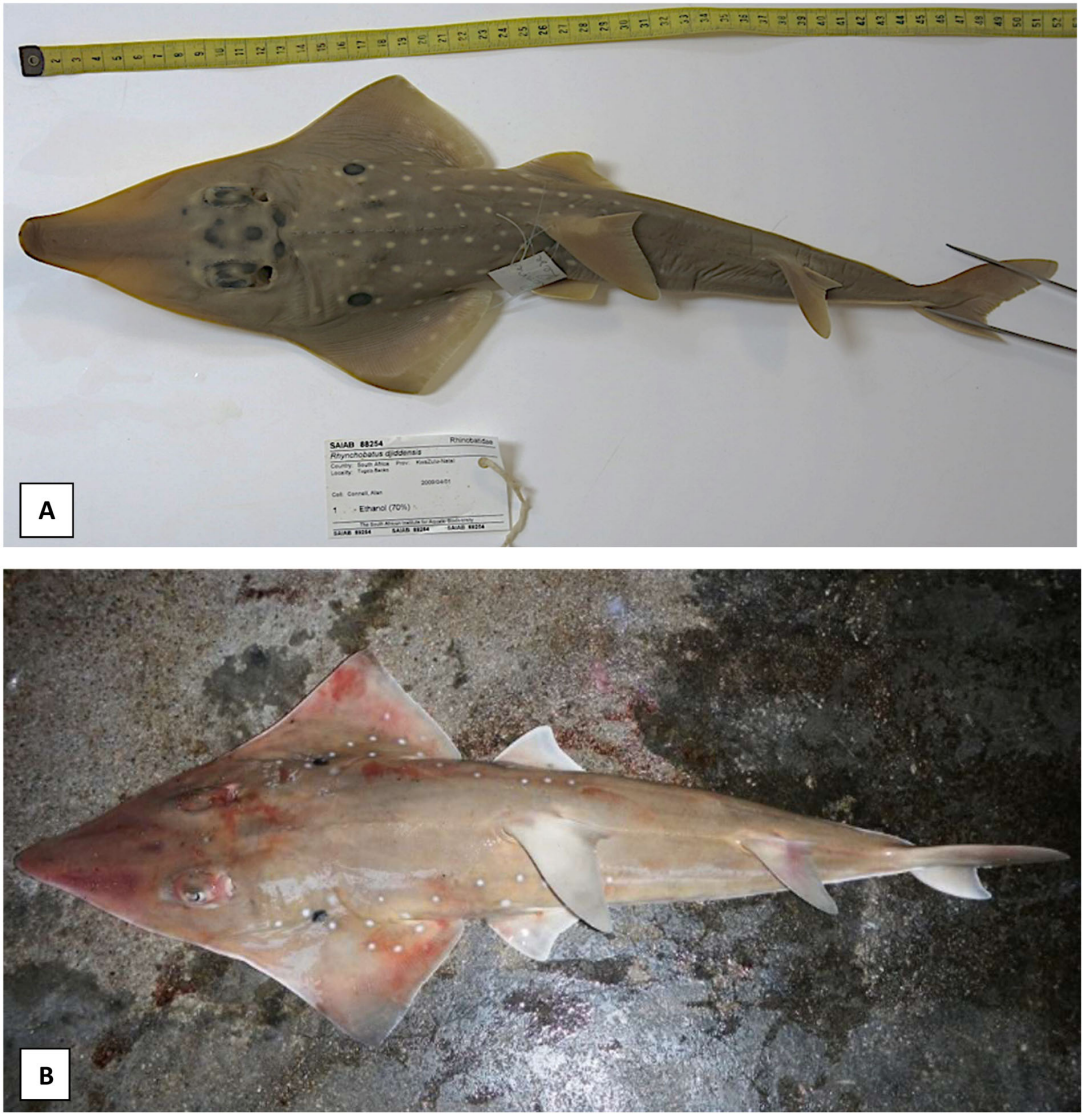
Species reference images of (A) *Rhynchobatus djiddensis* (Photo by the South African Institute for Aquatic Biodiversity), the whitespotted wedgefish, with prominent black markings between eyes, large number of white spots and black pectoral marking surrounded by four or more white spots and (B) *Rhynchobatus australiae* (Photo by John Nevill), the bottlenose wedgefish, with bottle-shaped snout slightly constricted near tip, three white spots aligned over the pectoral marking (usually two spots below), a short line of well-demarcated white spots on the mid dorsal surface and no spots on the tail (TL = 137 cm).

In this study, the *R. djiddensis* specimen was collected in Sodwana Bay, KwaZulu-Natal, South Africa (27.5565° S, 32.6673° E; sample ID: SALS-050.2) and the *R. australiae* specimenin Unguja, Zanzibar, Tanzania (6.1357° S, 39.3621° E; sample ID: FID7731). The specimens were morphologically identified (please see Figure 1 for references images) and confirmed by molecular species identification based on the cytochrome oxidase c subunit 1 (*COX1*) and nicotinamide adenine dinucleotide hydride dehydrogenase subunit 2 (*NADH2*). Fin-clip samples and DNA are stored at the Genetics Department of Stellenbosch University, South Africa (http://www.sun.ac.za/english/faculty/agri/genetics, Aletta Bester-van der Merwe, aeb@sun.ac.za). Total genomic DNA was extracted from fin-clip samples using a standard cetyltrimethylammonium bromide extraction protocol (Sambrook and Russell 2001). The quality and quantity were assessed using a NanoDrop™ ND 2000 spectrophotometer (Thermo Fisher Scientific, Waltham, MA). Low-coverage whole-genome sequencing was performed on an Ion Torrent S5™ System at the Central Analytical Facility at Stellenbosch University, South Africa. All sequencing reads were quality filtered using Torrent Suite™ Software.

### Mitogenome assembly, phylogenetic reconstruction, and variation analysis

Ion Torrent reads were mapped to the previously published mitogenome of *R. australiae* (KU746924) using the Geneious Read Mapper algorithm with default parameters (Kearse et al. 2012). The consensus sequences were annotated with the web-based tool MitoAnnotator (Iwasaki et al. 2013) and the Sequence Manipulation Suite (Stothard 2000) was used to check for translation errors. The annotated mitogenome sequences can be accessed via GenBank under accession numbers ON065568 for *R. djiddensis* and ON065567 for *R. australiae*. The mitogenomes were drawn into full circular genome with Proksee (https://proksee.ca) which uses CGView.js as its genome drawing engine (Grant and Stothard 2008). Strand asymmetry was measured based on the following formulas: AT skew = (A – T)/(A + T) and GC skew = (G – C)/(G + C) (Perna and Kocher 1995).

We inferred the phylogenetic placement of *R. djiddensis* and *R. australiae* by aligning the mitogenomes against 13 whole mitogenome sequences from the order Rhinopristiformes (Table 1), using MAFFT (Katoh and Standley 2013) with the L-INS-i algorithm on Geneious (Kearse et al. 2012). Two mitogenomes from Arhynchobatidae and three from Rajidae were included as outgroups. The alignment was based on 13 concatenated protein-coding genes of each mitogenome. The nucleotide substitution models that best fitted the alignment were determined in PhyloSuite using PartitionFinder2 (Lanfear et al. 2016) according to the Akaike’s information criterion with correction for small sample size (AICc) as recommended by Lanfear et al. (2016). Sequences were partitioned into coding domain sequences (CDS) to account for the divergent phylogenies of sequence regions undergoing unique evolution. Maximum-likelihood (ML) inference of the phylogenetic relationships among mitogenomes was performed using IQ-TREE (Nguyen et al. 2015) with 1000 bootstrap replicates and Bayesian inference (BI) was performed in MRBAYES v3.2.7a (Ronquist et al. 2012) with 2,000,000 MCMC generations and the first 500,000 generations discarded as burn-in. The consensus tree was visualized with The Interactive Tree Of Life (iTOL) v5 (Letunic and Bork 2021). Additionally, the mitogenomes from this study and the online *R. australiae* sequence (KU746924) were aligned in Geneious as previously described, whereafter Mega 11 (Tamura et al. 2021) was used to identify the most variable regions across all protein-coding genes and the control region between the species.

**Table 1.** Whole mitogenomes from the order Rhinopristiformes used to infer the phylogenetic placement of *R. djiddensis* (ON065568) and *R. australiae* (ON065567).

Family	Species	Size (bp)	GenBank accession number	Reference
Glaucostegidae	*Glaucostegus granulatus* (Cuvier, 1829)	16,547	MN783017	Johri, Fellows, et al. (2020)
Pristidae	*Anoxypristis cuspidata* (Latham, 1794)	17,243	KP233202	Chen, Kyne, et al. (2016)
	*Pristis clavata* (Garman, 1906)	16,804	KF381507	Feutry et al. (2015)
	*Pristis pectinata* (Latham, 1794)	16,802	KP400584	Chen, Wiley, et al. (2016)
	*Pristis pectinata* (Latham, 1794)	16,803	MF682494	Díaz-Jaimes et al. (2018)
	*Pristis pristis* (Linnaeus, 1758)	16,912	MH005928	Kyne et al. (2018)
	*Pristis zijsron* (Bleeker, 1851)	16,804	MH005927	Direct submission
Rhinidae	*Rhina ancylostoma* (Bloch & Schneider, 1801)	17,217	KU721837	Si, Chen, et al. (2016)
	*Rhynchobatus australiae* (Whitley, 1939)	16,804	KU746824	Si, Ding, et al. (2016)
	***Rhynchobatus australiae* (Whitley, 1939)**	**16,805**	**ON065567**	**This study**
	***Rhynchobatus djiddensis* (Forsskål, 1775)**	**16,799**	**ON065568**	**This study**
	*Rhynchobatus laevis* (Bloch & Schneider, 1801)	16,560	MN988687	Johri, Tiwari, et al. (2020)
Rhinobatidae	*Rhinobatos hynnicephalus* (Richardson, 1846)	16,776	KF534708	Chen et al. (2015)
	*Rhinobatos schlegelii* (Müller & Henle, 1841)	16,780	KJ140136	Chen, Ai, et al. (2016)
Trygonorrhinidae	*Zapteryx exasperata* (Jordan & Gilbert, 1880)	17,310	KM370325	Castillo-Páez et al. (2014)

Those in bold are the mitogenomes from this study.

## Results

**Figure 2.**
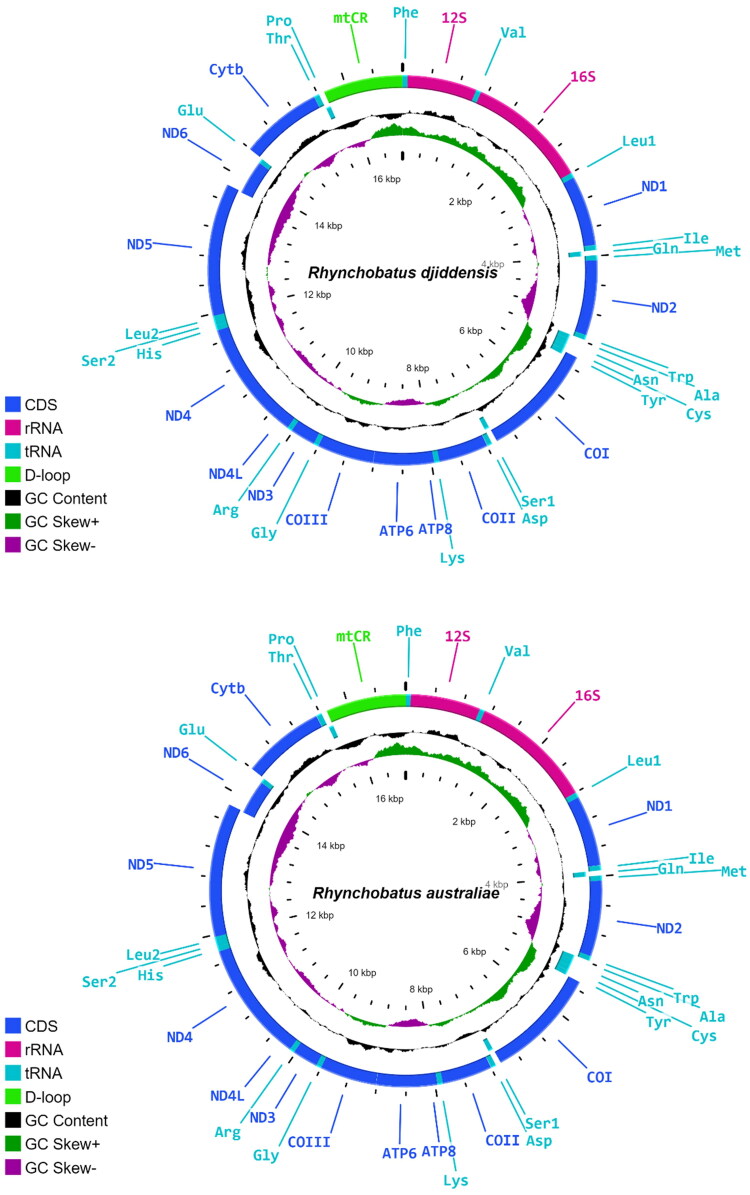
Gene maps of the *Rhynchobatus djiddensis* (ON065568) and *Rhynchobatus australiae* (ON065567) mitogenomes. The outermost circle genes are transcribed clockwise and the rest counter clockwise.

The obtained mitogenomes for *R. djiddensis* and *R. australiae* comprised 16,799 and 16,805 nucleotides, respectively (Figure 2). The overall base composition of the *R. djiddensis* genome was A: 32.3%, T: 27.7%, C: 26.7%, and G: 13.3%; and of the *R. australiae* genome was A: 32.3%, T: 27.6%, C: 26.9%, and G: 13.2%. Protein-coding gene regions were highly conserved between species, as commonly seen in elasmobranchs (Díaz-Jaimes et al. 2016).

**Figure 3.**
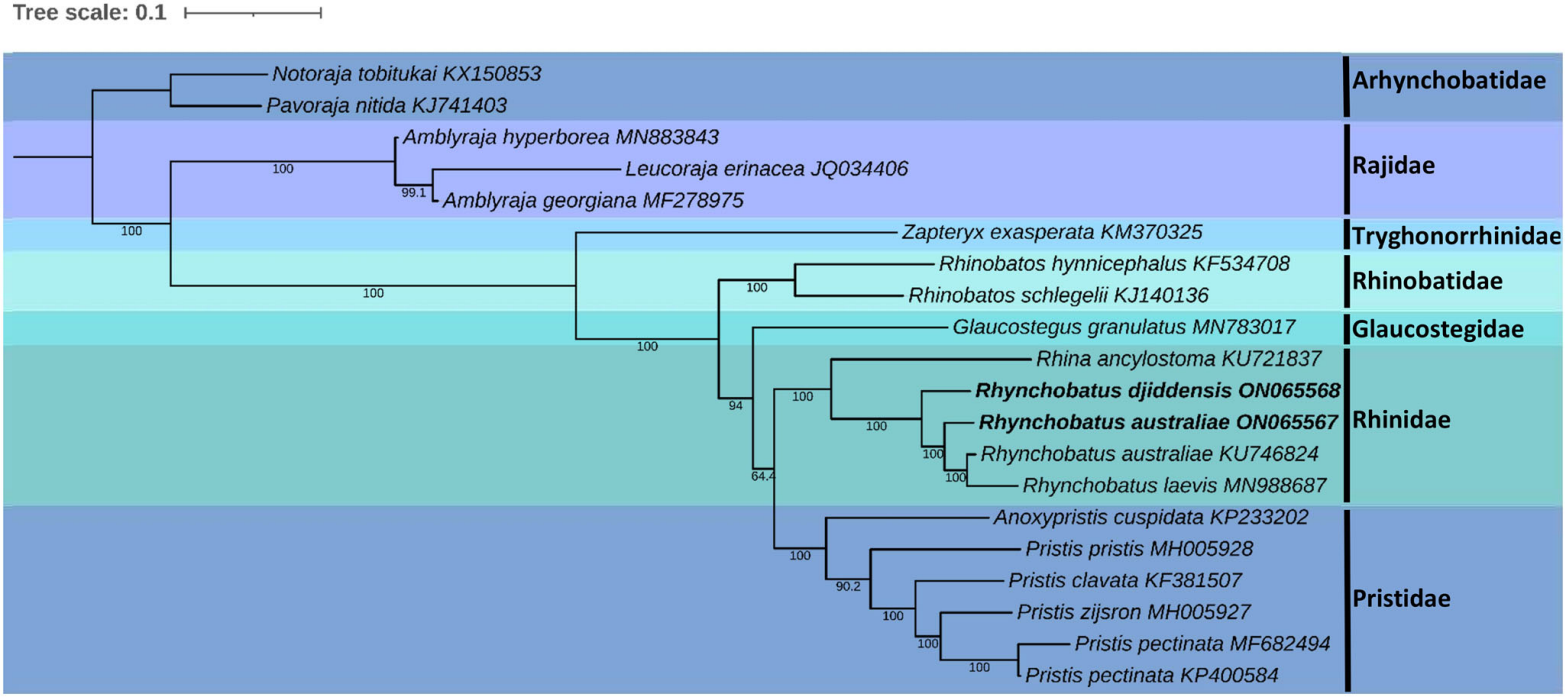
Maximum-likelihood phylogeny of five families from the order Rhinopristiformes based on the concatenated dataset of 13 protein-coding genes, with members of the families Rajidae and Arhynchobatidae included as outgroups. Numbers at nodes indicate bootstrap values. GenBank accession numbers are given adjacent to the species name, scale bar indicates groupings of species into families and the taxa in bold are the mitogenomes from this study.

The phylogenetic reconstruction places *R. djiddensis* and *R. australiae* within the genus *Rhynchobatus*, with the closest relationship to the other two wedgefish species, *R. laevis* and *R. australiae* (Figure 3). In addition to the placement of our specimens, the previously reported sample of *R. laevis* (Johri, Tiwari, et al. 2020) clusters within *R. australiae* with strong support in our ML analysis. Johri, Tiwari, et al. (2020), however, used *NADH2* genes to assess the phylogenetic placement of *R. laevis* where it also resided within the clade representing *Rhynchobatus*, but separate to other sister species. In our BI analysis, *R. laevis* clusters the same as in Johri, Tiwari, et al. (2020), with slightly less statistical support than our ML analysis (Figure S1).

The number of single nucleotide polymorphisms (SNPs) per gene region, and relative variation accounting for gene length, are listed in Table 2. A total of 481 SNPs were detected between the species (ambiguous sites were not included). *NADH5* was the most variable in terms of total number of SNPs (75), followed by *COX1* (53) and *NADH2* (47). Taking gene length into account, *NADH2* was most variable (4.493%), followed by *NADH6* (4.239%) and *NADH5* (4.052%), with the control region ranking 7th (3.7%).

**Table 2.** Single nucleotide polymorphisms (SNPs) found in 16 regions of the mitogenomes from this study; *Rhynchobatus djiddensis* (ON065568) and *Rhynchobatus australiae* (ON065567), and the online-available *R. australiae* sequence (KU746924).

Gene	Length (bp)	SNPs	Relative variation (%)
*COX1*	1557	53	3.404
*COX2*	691	18	2.605
*COX3*	786	25	3.181
*NADH1*	975	38	3.897
*NADH2*	1046	47	4.493
*NADH3*	351	9	2.564
*NADH4L*	297	11	3.704
*NADH4*	1381	38	2.752
*NADH5*	1851	75	4.052
*NADH6*	519	22	4.239
*CYTB*	1143	46	4.024
*12S rRNA*	967	7	0.724
*16S rRNA*	1690	24	1.420
*ATP6*	684	22	3.216
*ATP8*	168	5	2.976
Control region	1108	41	3.700

ATP: adenosine triphosphate; CO: cytochrome c oxidase subunit; CYTB: cytochrome b; NADH: nicotinamide adenine dehydrogenase subunit; rRNA: ribosomal ribonucleic acid.

## Discussion

There is a pronounced need for updated and accurate genomic resources, particularly in the marine environment. Advances in high-throughput sequencing technologies have led to the development of genomic resources with great potential for applications in marine conservation, yet large-scale sequence information for elasmobranchs remains relatively scarce with limited genetic resources especially for batoids (Pearce et al. 2021). In this study, the use of low-coverage whole-genome sequencing data enabled the development of molecular resources, namely the complete mitogenomes of *R. djiddensis* and *R. australiae*. While our phylogenetic reconstruction supports the monophyly of *Rhynchobatus* and finds similar relationships as previous studies (Si, Ding, et al. 2016; Johri, Fellows, et al. 2020; Kousteni et al. 2021), full resolution of the order is limited by the lack of available data. Future studies should focus on generating and analyzing whole mitochondrial genomes for batoids, which is more attainable than a few years ago due to recent technological advancements. Additionally, the previously reported sample of *R. laevis* (Johri, Tiwari, et al. 2020) grouped within *R. australiae* in our ML analysis but separately in our BI analysis. Phylogenetic uncertainty translates to taxonomic uncertainty (Chafin et al. 2021), and the latter is significantly associated with the *Rhynchobatus* genus. Our mitogenome analysis also highlights areas of variability to serve as a guideline for locating and designing primers to target the most informative mitochondrial regions in *R. djiddensis* and *R. australiae*, with *NADH2* being the most variable. This challenges the suitability, at least within the *Rhynchobatus* genus, of the more conventional control region and *COX1* as standard mitochondrial markers for population studies or barcoding of elasmobranch species (Feutry et al. 2014; Klein et al. 2019). Our findings can be used to resolve misidentification issues and taxonomic disputes within the whitespotted wedgefish complex, as it is a prerequisite to the efficient implementation of conservation efforts and associated management based on elasmobranch population structuring patterns (Dudgeon et al. 2012; Bester-van der Merwe and Gledhill 2015). The taxonomic uncertainty within the *Rhynchobatus* genus is highlighted; therefore, these genomic resources in combination with those already developed, can support the sustainable management of wedgefishes using a precautionary approach.

## Supplementary Material

Supplemental Material

Supplemental Material

Supplemental Material

## Data Availability

The genome sequence data that support the findings of this study are openly available in GenBank of NCBI (https://www.ncbi.nlm.nih.gov/) under the accession numbers ON065567–ON065568. The associated BioProject, SRA, and Bio-Sample numbers are PRJNA886704, SRX17782697–SRX17782698, and SAMN31139799–SAMN31139800, respectively.
