# Supplementary material for: Characterization of the complete mitochondrial genomes of two Critically Endangered wedgefishes: Rhynchobatus djiddensis and Rhynchobatus australiae
Source: Mitochondrial DNA B Resour. 2023 Mar 7;8(3):352–8. doi: 10.1080/23802359.2023.2167479 (PMC10013529; doi:10.1080/23802359.2023.2167479)
Supplement: Supplemental Material [file TMDN_A_2167479_SM5077.pdf]

Run Summary

6.26 G
Total Bases

98
Key Signal

93%
ISP Loading

ISP Density

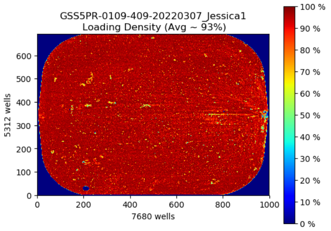

19,737,610
Total Reads

56%
Usable Reads

ISP Summary

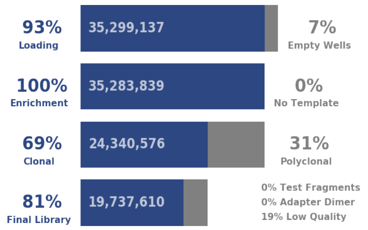

317 bp
Mean

359 bp
Median

382 bp
Mode

Read Length

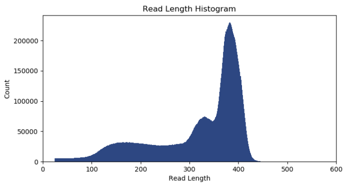

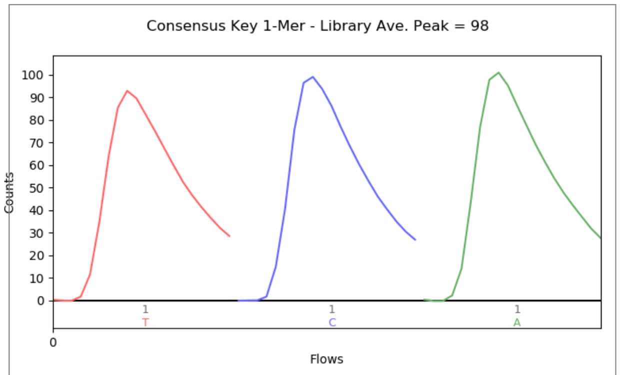

**Addressable Wells**	**37,849,615**	
With ISPs	35,299,137	93.3%
Live	35,283,839	100.0%
Test Fragment	109,887	00.3%
Library	35,173,952	99.7%

**Library ISPs**	**35,173,952**	
Filtered: Polyclonal	10,943,263	31.1%
Filtered: Low Quality	4,457,624	12.7%
Filtered: Adapter Dimer	35,455	00.1%
**Final Library ISPs**	**19,737,610**	**56.1%**

Barcode Name	Sample	Bases	$\geq Q20$	Reads	Mean Read Length	Read Length Histogram
No barcode	none	281,434,430	255,324,463	848,240	331 bp	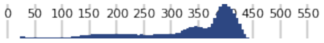
IonCode_0189	SALS-050.2_Rhynchobatus_djiddensis	3,517,044,026	3,188,240,436	11,375,920	309 bp	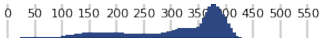
IonCode_0190	7731_Rhynchobatus_australiae	2,466,029,104	2,240,270,913	7,513,062	328 bp	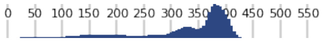

Test Fragment	Reads	Percent 50AQ17	Read Length Histogram
**TF_1**	**27,825**	**92**	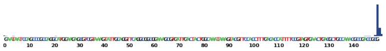

iontorrent by Thermo Fisher Scientific

Alignment Summary *(aligned to)*

0	
Total Alignment Bases	Average Coverage Depth of Reference

alignment_rate_plot.png

	Count	%
**Total Reads**	0	–
**Aligned Reads**	0	%

%

Mean Raw Accuracy 1x

base_error_plot.png

Alignment Quality

	AQ17	AQ20	Perfect
**Total Number of Bases [Mbp]**	0	0	0
**Mean Length [bp]**	0	0	0
**Longest Alignment [bp]**	0	0	0
**Mean Coverage Depth**			

Filtered_Alignments_Q10.png

Filtered_Alignments_Q17.png

Filtered_Alignments_Q20.png

Filtered_Alignments_Q47.png

Analysis Details

**Run Name**	R_2022_03_08_09_37_00_user_GSS5PR-0109-409-20220307_Jessica1
**Run Date**	March 8, 2022, 9:38 a.m.
**Run Flows**	850
**Projects**	
**Samples**	SALS-050.2_Rhynchobatus_djiddensis, 7731_Rhynchobatus_australiae
**Reference**	
**Instrument**	GSS5PR-0109
**Operation Mode**	Customer mode
**Flow Order**	TACGTACGTCTGAGCATCGATCGATGTACAGC
**Library Key**	TCAG
**TF Key**	ATCG
**Chip Barcode**	DAHF00125
**Chip Check**	Passed
**Chip Type**	530
**Chip Data**	tiled
**Chip Lot Number**	Q1XP12
**Chip Wafer**	03
**Barcode Set**	IonCode
**Analysis Name**	Auto_user_GSS5PR-0109-409-20220307_Jessica1_768
**Analysis Date**	March 8, 2022, 4:37 p.m.
**Analysis Flows**	0
**runID**	ISTCB
**BeadFind Args**	justBeadFind –args-json /opt/ion/config/args_530_beadfind.json
**Analysis Args**	Analysis –args-json /opt/ion/config/args_530_analysis.json
**Pre-BaseCaller**	BaseCaller –trim-qual-cutoff 15 –barcode-filter-minreads 10 –phasing-residual-filter=2.0 –wells-normalization on
**Calibration Args**	Calibration –num-calibration-regions 1,1
**BaseCaller Args**	BaseCaller –trim-qual-cutoff 15 –barcode-filter-minreads 10 –phasing-residual-filter=2.0 –num-unfiltered 1000 –barcode-filter-postpone 1 –qual-filter true –qual-filter-slope 0.040 –qual-filter-offset 1.0 –wells-normalization on
**Alignment Args**	tmap mapall -q 50000 ... stage1 map4
**IonStats Args**	ionstats alignment
**Analysis Parameters**	default

Chef Summary

Chef Template Prep Information:

**Chef Last Updated**	March 8, 2022, 9:02 a.m.
**Chef Instrument Name**	CHEF00400
**Chef Operation Mode**	Customer Mode
**Sample Position**	1
**Tip Rack Barcode**	4B7220003
**Chip Type 1**	530v1
**Chip Type 2**	530v1
**Chip Expiration 1**	None
**Chip Expiration 2**	None
**Templating Kit Type**	Ion 510
amp; Ion 520	amp; Ion 530 Kit-Chef
**Chef Flexible Workflow**	
**Reagent Expiration**	220630
**Reagent Lot Number**	2355382
**Reagent Part Number**	A34018C
**Reagent Cartridge Serial Number**	None
**Solution Lot Number**	2307512
**Solution Part Number**	A27754C
**Templating Protocol Planned**	Chef Protocol - 400 bp
**Solution Cartridge Serial Number**	None
**Solution Expiration**	220430
**Templating Protocol Executed**	Chef Protocol - 400 bp
**Chef Script Version**	2005
**Chef Package Version**	IC.5.16.1
**Start Time**	March 7, 2022, 6:21 p.m.
**Completion Time**	March 8, 2022, 9:02 a.m.

S5 Consumables Summary

**Chip Type**	530v1
**Chip Barcode**	DAHF00125

Product Description	Part Number	Lot Number	Exp. Date	Remaining Uses
Ion S5 Cleaning Solution	100031096	2344780	2022/11/30	3
Ion S5 Sequencing Reagent	INS1012841B	2322829	2022/06/30	1
Ion S5 Wash Solution	100031091B	2346448	2022/09/30	1

Software Version

**Torrent_Suite**	5.16.1
**host**	GVW90Q2
**ion-analysis**	5.16.6-1
**ion-dbreports**	5.16.28-1
**ion-gpu**	5.16.1-1
**ion-pipeline**	5.16.14-1
**ion-torrentpy**	5.16.6-1
**ion-torrentr**	5.16.6-1
**S5 Script**	0.1.32
**LiveView**	2762
**DataCollect**	3976
**OIA**	51231
**OS**	35
**Graphics**	134
**Ion_Chef**	IC.5.16.1
