# Supplementary material for: Characterization of the complete mitochondrial genomes of two Critically Endangered wedgefishes: Rhynchobatus djiddensis and Rhynchobatus australiae
Source: Mitochondrial DNA B Resour. 2023 Mar 7;8(3):352–8. doi: 10.1080/23802359.2023.2167479 (PMC10013529; doi:10.1080/23802359.2023.2167479)
Supplement: Supplemental Material [file TMDN_A_2167479_SM5073.pdf]

Report: Genomic DNA (gDNA) Quality Control

CAF: Next Generation Sequencing Division

UNIVERSITEIT STELLENBOSCH UNIVERSITY

Project Number Proj2022_003b
CAF User: Mia Groeneveld
CAF ID: CAF-21-30127

Tube #	Sample Name	QC date	Nanodrop (ng/ul)	260/280	260/230	Pass QC Nanodrop [1]	Qubit (ng/ul)	Volume after QC	Pass QC Amount [2]	GQS	Pass QC Integrity [3]	Recommendation[4]
1	SALS-050.2_Rhynch	2022/01/28	812.1	1.95	2.37	PASS	404	35	PASS	4.7	PASS	Library construction
2	SALS-154_Rhynchob	2022/01/28	275.4	1.95	2.54	PASS	125	35	PASS	4.2	PASS	Library construction
3	7731_Rhynchobatus	2022/01/28	24.6	2.12	2.36	PASS	6.9	35	PASS	3.1	PASS	Library construction
4	6764_Rhynchobatus	2022/01/28	22.2	1.89	3.68	PASS	3	35	PASS	3.8	PASS	Library construction
5	E100_Triakis_megal	2022/01/28	64.9	2	2.59	PASS	24	35	PASS	4.5	PASS	Library construction
6	CPW1_Triakis_mega	2022/01/28	134.6	2.01	2.51	PASS	73.2	35	PASS	4.4	PASS	Library construction
7	Mpa#51_Mustelus_	2022/01/28	368.2	1.96	2.24	PASS	110	35	PASS	**0.6**	FAIL	Re-submit
8	Mpa#53_Mustelus_	2022/01/28	46	1.93	2.64	PASS	10.6	35	PASS	**1.2**	FAIL	Re-submit

Key: GQS - The Genome Quality Score (GQS) is determined by electrophoresis using the PerkinElmer LabChip and ranges between 0 and 5, with zero indicating the lowest and five, the highest quality , intact gDNA.
[1] **PASS**: The gDNA sample is of a high quality, with a 260/280 ratio ranging between 1.8 and 2.2, and ideally a 260/230 ratio $\geq$ 1.0. **FAIL**: The gDNA sample is of a lower quality, with a 260/280 ratio outside the 1.8 - 2.2 range and/or a 260/230 ratio < 1.
[2] **PASS**: Sufficient gDNA (>100ng) is available at a minimum concentration of 0.76ng/ul, as determined by fluorometry, for library construction. **FAIL**: Less than 100ng gDNA is available and/ or the dsDNA concentration is less than 0.76ng/ul, as determined by fluorometry, for library construction.
[3] **PASS**: The gDNA sample consists primarily of high molecular weight dsDNA with a GQS between 3 and 5. **FAIL**: The gDNA sample consists primarily of sheared DNA and as a result, has a GQS below 3.
[4] As a part of the NGS service at the CAF we offer gDNA sample purification. Note that, depending on the level of protein, salt and organic solvent contamination, up to 80% of the sample material may be lost and as such, might result in insufficient material for library construction.
