# Supplementary material for: Characterization of the complete mitochondrial genomes of two Critically Endangered wedgefishes: Rhynchobatus djiddensis and Rhynchobatus australiae
Source: Mitochondrial DNA B Resour. 2023 Mar 7;8(3):352–8. doi: 10.1080/23802359.2023.2167479 (PMC10013529; doi:10.1080/23802359.2023.2167479)
Supplement: Supplemental Material [file TMDN_A_2167479_SM5076.pdf]

UNIVERSITEIT
STELLENBOSCH
UNIVERSITY

Approved with Stipulations

29 March 2021

PI: Prof Aletta Van Der Merwe

REC: ACU Reference #: ACU-2021-21616

Title: Towards an Improved Biodiversity Assessment and Management of Heavily Exploited Elasmobranchs in South Africa.

Dear Prof Aletta Van Der Merwe

Your Animal Notification, with reference number #ACU-2021-21616 was reviewed on 17 March 2021 by the Research Ethics Committee: Animal Care and Use via committee review procedures and was approved on condition that the following stipulations are adhered to:

1. How was the animals killed: Applicant must revise this section to: As killed by fishermen or natural causes.

Committee Suggestion - Disposal of material: specimens should be preserved to confirm identification.

Applicants are reminded that they are expected to comply with accepted standards for the use of animals in research and teaching as reflected in the South African National Standards 10386: 2008. The SANS 10386: 2008 document is available on the Division for Research Developments website www.sun.ac.za/research.

As provided for in the Veterinary and Para-Veterinary Professions Act, 1982. It is the principal investigator's responsibility to ensure that all study participants are registered with or have been authorised by the South African Veterinary Council (SAVC) to perform the procedures on animals, or will be performing the procedures under the direct and continuous supervision of a SAVC-registered veterinary professional or SAVC-registered para-veterinary professional, who are acting within the scope of practice for their profession.

Please remember to use your REC: ACU reference number: # ACU-2021-21616 on any documents or correspondence with the REC: ACU concerning your research protocol.

If you have any questions or need further help, please contact the REC: ACU office at 021 808 9003.

Visit the Division for Research Developments website www.sun.ac.za/research for documentation on REC: ACU policy and procedures.

Sincerely,

Mr Winston Beukes

Coordinator: Research Ethics (Animal Care and Use)
